# Chorismate synthase mediates cerebral malaria pathogenesis by eliciting salicylic acid-dependent autophagy response in parasite

**DOI:** 10.1242/bio.054544

**Published:** 2020-12-21

**Authors:** Malabika Chakrabarti, Deepika Kannan, Akshay Munjal, Hadi Hasan Choudhary, Satish Mishra, Shailja Singh

**Affiliations:** 1 Host-Parasite Interaction & Disease Modelling Laboratory, Special Center for Molecular Medicine, Jawaharlal Nehru University, New Delhi-110067, India; 2 Department of Life Science, Shiv Nadar University, Noida, UP 201314, India; 3 Division of Parasitology, CSIR-Central Drug Research Institute, Lucknow 226031, India

**Keywords:** Cerebral malaria, Salicylic acid, Chorismate synthase, *Plasmodium falciparum*, *Plasmodium berghei* ANKA

## Abstract

Cerebral malaria caused by *Plasmodium falciparum* is the severest form of the disease resulting in the morbidity of a huge number of people worldwide. Development of effective curatives is essential in order to overcome the fatality of cerebral malaria. Earlier studies have shown the presence of salicylic acid (SA) in malaria parasite *P. falciparum*, which plays a critical role in the manifestation of cerebral malaria. Further, the application of SA for the treatment of acute symptoms in cerebral malaria increases the activity of iNOS leading to severe inflammation-mediated death, also called as Reye's syndrome. Therefore, modulation of the level of SA might be a novel approach to neutralize the symptoms of cerebral malaria. The probable source of parasite SA is the shikimate pathway, which produces chorismate, a precursor to aromatic amino acids and other secondary metabolites like SA in the parasite. In this work, we performed the immunological, pathological and biochemical studies in mice infected with chorismate synthase knocked-out *Plasmodium berghei* ANKA, which does not produce SA. Fewer cerebral outcomes were observed as compared to the mice infected with wild-type parasite. The possible mechanism behind this protective effect might be the hindrance of SA-mediated induction of autophagy in the parasite, which helps in its survival in the stressed condition of brain microvasculature during cerebral malaria. The absence of SA leading to reduced parasite load along with the reduced pathological symptoms contributes to less fatality outcome by cerebral malaria.

## Abstract (precis)

**Summary:** Our study identifies the role of parasite chorismate synthase and salicylic acid on host immunopathology and disease manifestation in cerebral malaria and points towards a pathway associated with salicylic acid-mediated autophagy of the malaria parasite.

## INTRODUCTION

Incidence of malaria leads to fatal consequences all over the world in every year (Trampuz et al., 2003; Bartoloni and Zammarchi, 2012). According to WHO malaria report 2019, approximately 405,000 deaths occurred worldwide due to infection by malaria parasite (World Health Organization, 2019). Cerebral malaria is the severest form of the disease caused by *Plasmodium falciparum* where the blood–brain barrier of the infected host is damaged and neurological symptoms like fever, seizures, retinopathy and alteration in brainstem volume are observed followed by irreversible coma and death (Bartoloni and Zammarchi, 2012). Due to the damage in the blood–brain barrier, internal hemorrhage occurs along with swelling of brain endothelium and blockage of capillaries due to sequestration of infected erythrocytes in the brain microvasculature (Medana and Turner, 2006). The condition is further aggravated by the effect of pro-inflammatory cytokines that are induced due to parasite infection causing more damage to the brain tissue (Dunst et al., 2017; Hunt and Grau, 2003; John et al., 2008). Effective measures need to be taken in order to control the progress of cerebral malaria along with reduction of parasite burden from the host system.

Salicylic acid (SA) is an agent known to play critical immunomodulatory role in cerebral malaria. SA and its derivatives have anti-pyretic and anti-inflammatory properties mediated through inhibition of cyclooxygenase enzymes and reduced level of prostaglandins (Brune, 1983; Fadeyi et al., 2004). However, earlier studies documented an increased death rate of malaria infected patients (especially children) when salicylate and its derivatives had been applied for the treatment of inflammatory symptoms of cerebral malaria (Ball et al., 2004). Moreover, the level of brain damage also increased with persistent activity of pro-inflammatory cytokines, for example TNFα, IL6, IL1β and IFNγ (Dunst et al., 2017; Brown et al., 1999). Previous studies had also demonstrated the manifestation of Reye's syndrome in malaria affected children of sub-Saharan Africa, which is characterized by loss of consciousness, confusion, seizure, convulsion and hepatomegaly followed by coma. Application of SA or its derivative to cure the febrile symptoms of cerebral malaria causes increased expression and activity of iNOS (inducible nitric oxide synthase) mediated through IFNγ leading to oxidative damage of brain tissue by nitric oxide free radicles (Clark et al., 2003, 2001).

Despite its immunomodulatory response, recent studies have indicated the role of SA in many biological processes including autophagy. SA as a plant metabolite facilitates the developing of resistance to different biotic and abiotic stress through induction of autophagy (Zhang and Chen, 2011; Yoshimoto et al., 2009). Similarly, Aspirin, the prodrug of salicylate, is also known to potently induce autophagy by repressing the activity of mammalian EP300 acetyltransferases (Lee and Finkel, 2009). As a result, the concentration of intracellular acetyl CoA decreases, resulting in diminution of the acetylation of several autophagic, cellular proteins, at the same time inducing autophagic flux. Although the mechanism of autophagy and its integrated regulation is well illustrated in mammals, their presence in Plasmodium warrants further investigation. The literature also supports the ability of parasites to cope with the nutrient deprivation via inducing autophagy, which might be the probable strategy of survival of parasite in cerebral malaria condition (Mancio-Silva et al., 2017; Kroemer et al., 2010; Joy et al., 2018). Therefore, the extent of cerebral malaria pathogenesis might be altered through the blockage of parasite chorismate synthase as well as application of SA in a nutrient starved condition.

In this background, the role of SA in parasite growth and survival in malaria remains elusive. On the other hand, parasite SA has an immune-regulatory role in manifestation of cerebral malaria through prostaglandin (PGE2)-mediated mechanism (Matsubara et al., 2015). However, the direct effect of parasite produced SA on the host immune system as well as progression of the disease needs to be explored further. In this context, we attempted to find out the exact role of SA in cerebral malaria as a parasite metabolite as well as a therapeutic agent, assuming that parasite SA is produced from the precursor molecule chorismate, the downstream product of shikimate pathway present in parasite, which is functional in other organisms including plants, fungi and bacteria, and generates aromatic amino acids. The shikimate pathway enzymes are good drug targets as this pathway is absent in all metazoans including humans. Determining the impact of parasite shikimate pathway on the immunopathology of cerebral malaria in connection with SA and parasite survival would be an interesting area to explore for novel target identification and drug development.

**Fig. 1.**
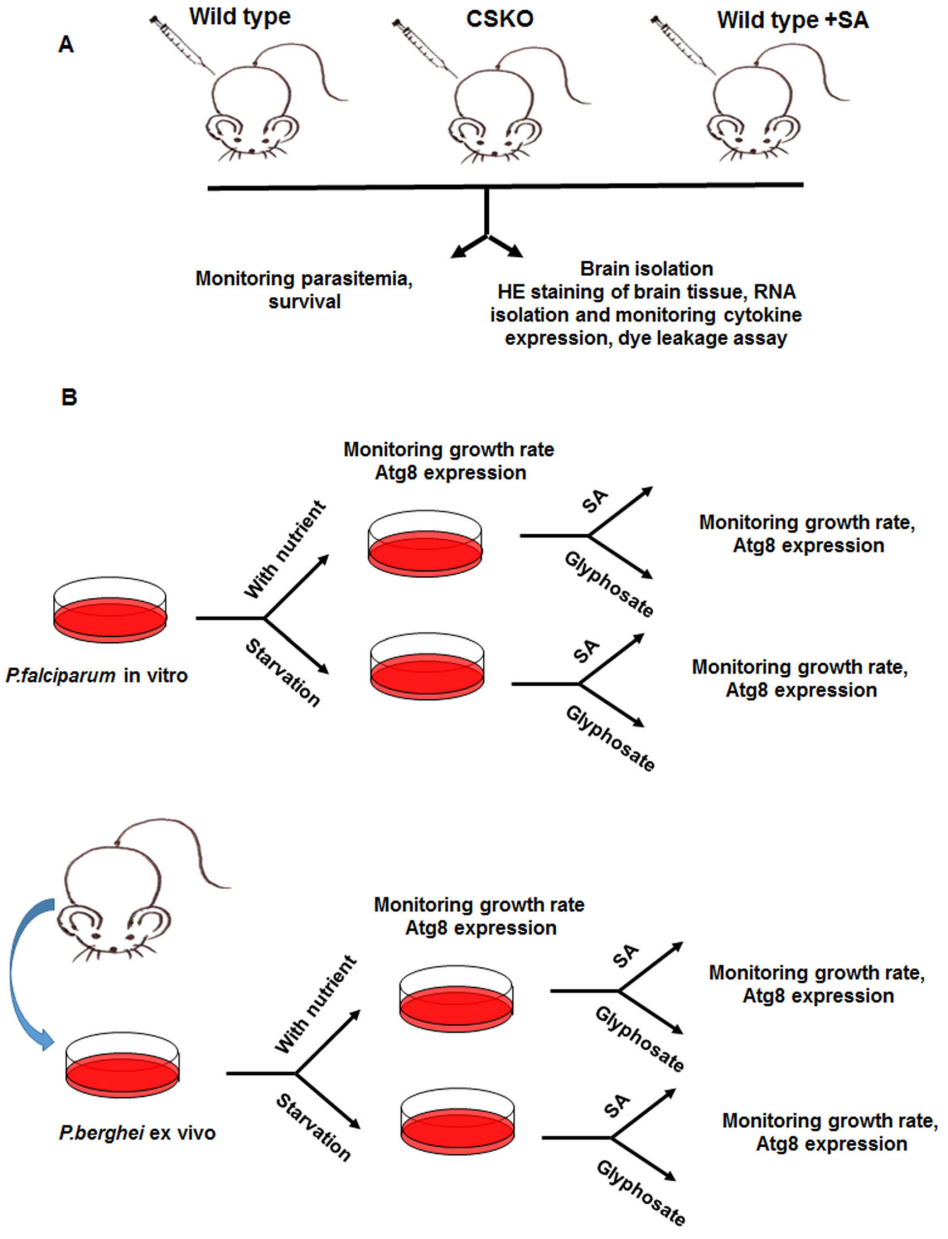
**Scheme of the experiments performed.** (A) Schematic showing the *in vivo* experimental plan for comparing the pathogenesis of wild-type and CS knockout *P. berghei*. (B) Schematic showing *in vitro* and *ex vivo* experiments with *P. falciparum* and *P. berghei*, respectively.

In this work, we monitored the survival, biochemical, pathological and immunological conditions of cerebral malaria mouse model infected with *Plasmodium berghei* ANKA in which chorismate synthase gene was knocked out (Choudhary et al., 2018) (PbA CSKO). The level of brain tissue and blood brain barrier damage was also measured (Fig. 1). Furthermore, we observed the effect of SA on *P. falciparum* autophagy and survival in normal and nutrient starved conditions (mimicking the stressed condition of the parasite in brain microvasculature in cerebral malaria). The Atg8 expression was evaluated in order to find out the possible mechanism behind SA-mediated autophagy in parasite and cerebral malaria pathogenesis and immunoregulation (Fig. 1).

## RESULTS

### Survival of mice infected with *P. berghei* wild type and CSKO

**Fig. 2.**
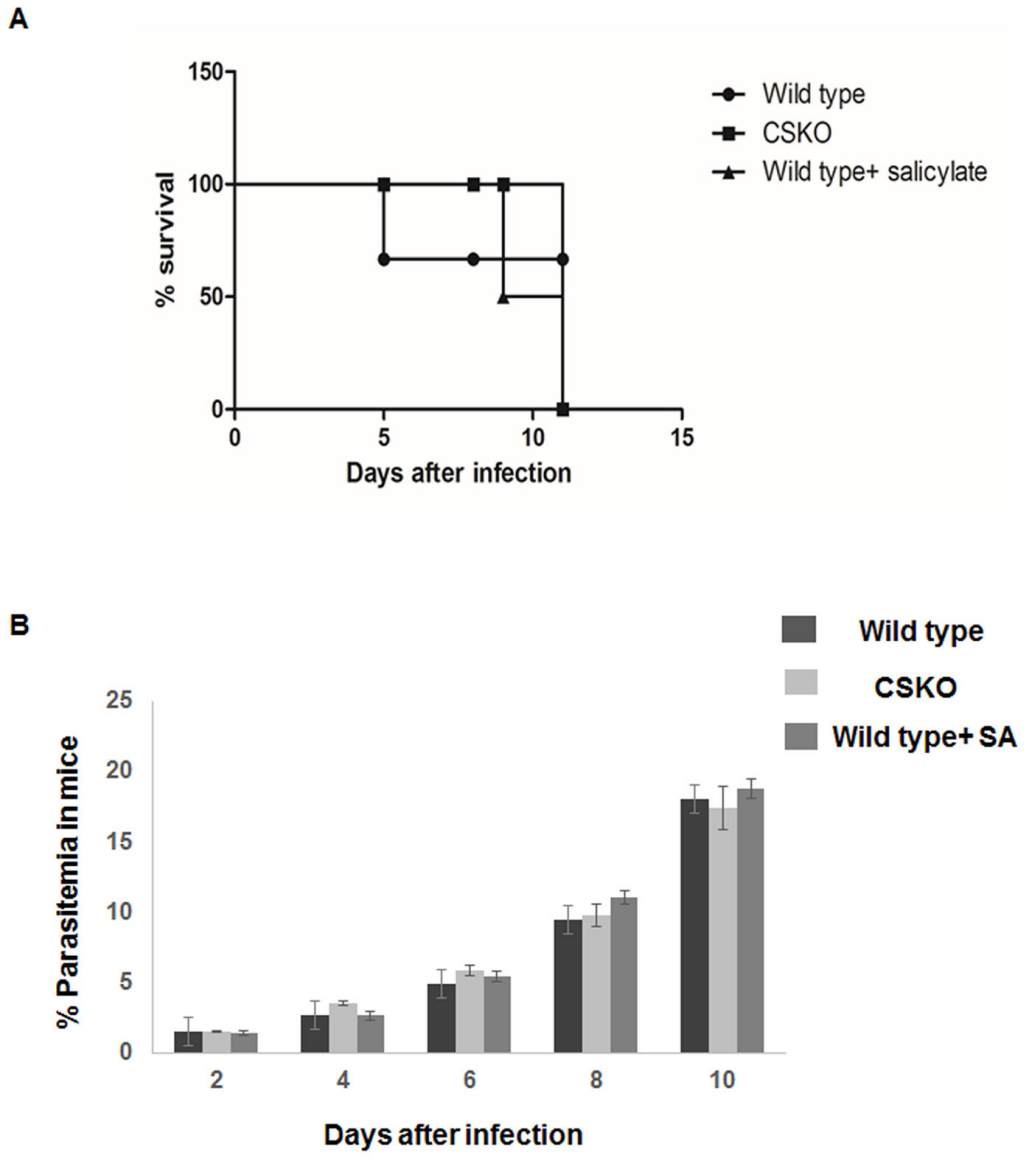
**Comparative study of survival of mice infected with *P. berghei* wild type and CS knockout**. (A) Graph showing percent survival of mice infected with *P. berghei* wild type along with SA treated and CS knockout. (B) Graph showing increasing parasitemia of the mice of the three experimental groups. Data are represented from two independent experiments.

Mice infected with *P. berghei* ANKA wild type and Chorismate synthase knockout were monitored until 12 days after infection. One group of mice infected with wild-type parasite was injected with SA on a daily basis. Parasitemia in the three groups of mice increased exponentially each day in a similar manner (Fig. 2B). Mice infected with wild type and treated with SA showed reduced survival as compared to the mice infected with CSKO parasite (Fig. 2A). On the eighth day after infection, parasitemia of all the mice reached around 10% and half of the mice died in the case of the wild-type PbA infection mice treated with and without SA, whereas mice infected with CSKO parasite were alive. On the twelfth day after infection, the survival percentage of wild-type infected mice treated was 50%. In the case of the wild-type parasite infected mice treated with SA, the survival percentage was 33%, whereas for knockout parasite infected mice, the survival rate was 83% (Fig. 2A). This result indicates reduced virulence of the CSKO parasite as compared to the wild-type parasite, with increased survival rate at high parasitemia. The experiment was performed with eight mice in each group.

### Comparison of brain tissue damage in mice infected with *P. berghei* ANKA wild type and CSKO

**Fig. 3.**
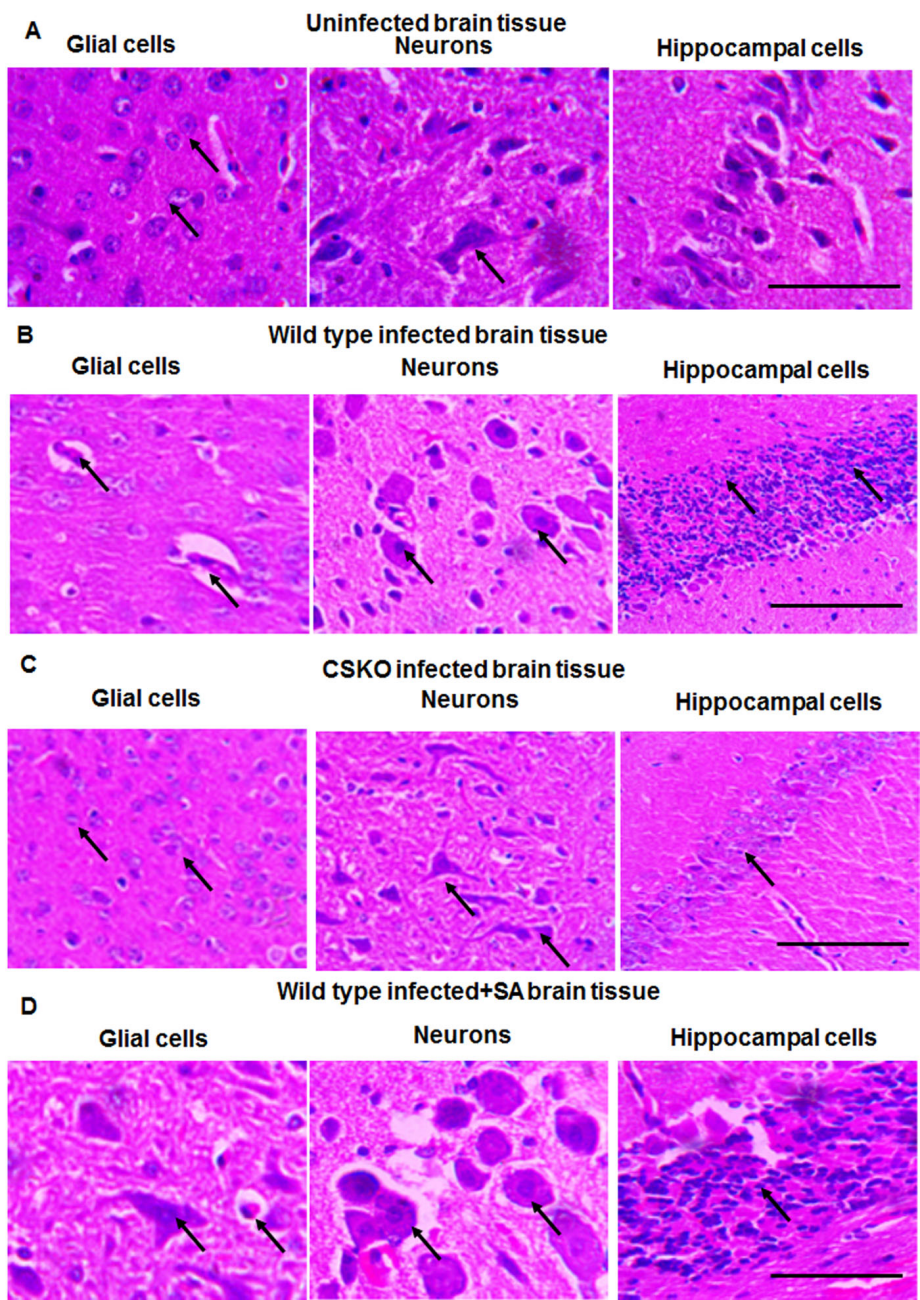
**Comparison of brain tissue damage in mice infected with *P. berghei* ANKA wild type and CSKO.** (A) Brain tissue sections showing neurons, glial cells and hippocampal region of uninfected mice. (B) Brain tissue sections showing neurons, glial cells and hippocampal region of mice infected with wild-type *P. berghei*. (C) Brain tissue sections showing neurons, glial cells and hippocampal region of mice infected with chorismate synthase knockout *P. berghei*. (D) Brain tissue sections showing neurons, glial cells and hippocampal region of mice infected with wild-type *P. berghei* along with SA treatment.

In order to monitor the level of brain damage caused by wild-type and knockout parasite, brain tissue sections were stained with Hematoxylin and Eosin and observed under microscope. In uninfected mice, the cell morphology was normal (Fig. 3A, indicated with black arrow) with healthy neurons and glial cells. Neuronal cells in the hippocampal region showed intact morphology. In case of mice infected with wild-type parasite, brain damage was observed with impaired blood vessel, damaged neurons with shrunken cytoplasm darkly stained with Eosin. Swelling of neurons was also observed (Fig. 3B). Cells in the hippocampal region were also shrunken with darkly stained pyknotic nuclei. Similar signs were found in mouse brain infected with wild-type parasite and treated with SA (Fig. 3D). On the other hand, the mice brain infected with knockout parasite showed somewhat normal morphology of cells which was more like uninfected control (Fig. 3C). The neuronal cells were healthy with normal morphology of hippocampal cells. Therefore, the lesser degree of brain cell damage and change in histopathology suggests decreased cerebral outcome due to infection of chorismate synthase knockout parasite. The brains of five mice from each group were used for sectioning and subsequent staining in case of two independent experiments in order to see the condition of brain tissue of significant number of mice. Scale bar in the images represents distance of 100 µm.

### Level of pro-inflammatory cytokines in mice infected with wild type and CSKO *P. berghei* ANKA

**Fig. 4.**
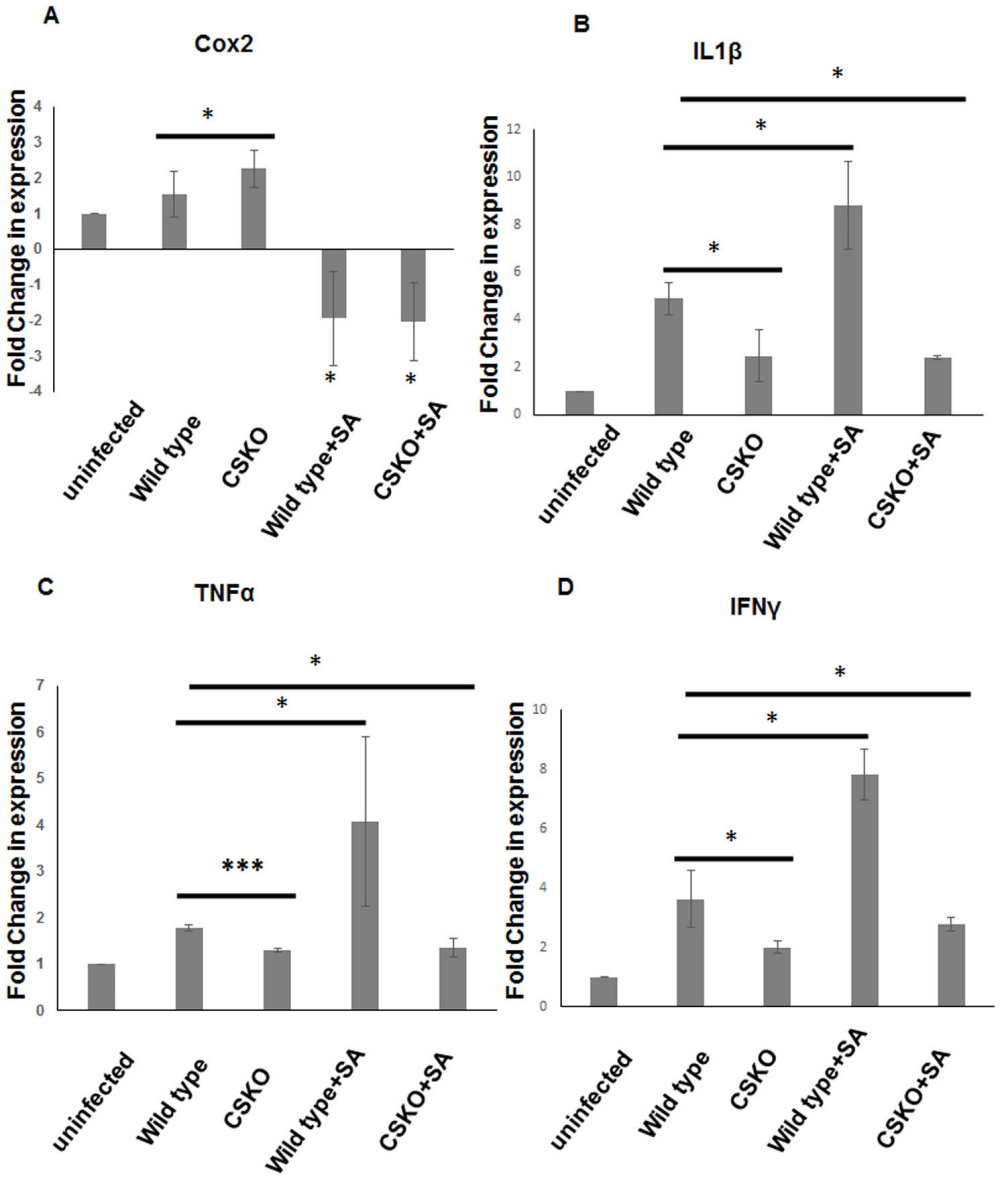
**Level of pro-inflammatory cytokines in groups of mice infected with wild-type and CSKO parasite along with the two groups infected with the wild-type and knockout parasite, respectively, and subsequently treated with SA.** Graphs showing transcriptional levels of the following genes in mouse brain total RNA, (A) Cox2, (B) IL1β, (C) TNFα, (D) IFNγ. Data are represented as mean±s.d. from two independent experiments.

Level of inflammation in the infected mice was observed by checking the expression pattern of Cox2 and pro-inflammatory cytokines IL1β, TNFα and IFN-γ eliciting inflammatory and febrile symptoms as well as activation of macrophages. Significant increase in Cox2 expression (*P<0.05) was observed in knockout parasite infected mice brain indicating more PGE2 production and stalling of the activity of pro-inflammatory cytokines after a certain period of time (Fig. 4A). A group of mice was included in this experiment that were infected with the knockout parasite and injected with salicylic acid till the 10th day of infection. In case of the wild type and CSKO parasite infected mice treated with SA, significant downregulation of cox2 was observed (Fig. 4A). The data show a pronounced effect of the application of SA on mice. On the other hand, significant reduction of the expression of pro-inflammatory cytokines is seen in CSKO knockout parasites IL1β (*P<0.05), TNFα (***P<0.001) and IFNγ (*P<0.05) (Fig. 4B–D) was observed as compared to the mice infected with the wild-type parasite showing decreased activation of immune system and oxidative damage mediated by iNOS, which is responsible for Reye's syndrome. The level of IL1β, TNFα and IFNγ was nearly twofold higher in the mice infected with the wild-type parasite and subsequently treated with SA in comparison with the ones infected with the wild-type parasite without SA treatment (Fig. 4B–D; *P*<0.05). For the mice infected with the knockout parasite with subsequent treatments of SA, the cytokine profile was similar to that of the mice infected with CSKO parasite. Moreover, the group showed a significant difference in the level of pro-inflammatory cytokine expression from the mice infected with the wild-type parasite (Fig. 4B–D; *P*<0.05). On this basis, it can be inferred that the application of SA in CSKO parasite-infected mice does not produce inflammatory response unlike the ones infected with the wild-type parasite. The reason behind this phenomenon might be the lack of SA in the CSKO parasite, resulting in less salicylate toxicity on the application of exogenous SA. Mouse GAPDH was used as a housekeeping gene for the analysis.

### Comparison of blood–brain barrier damage in wild type and CSKO parasite infected mice

**Fig. 5.**
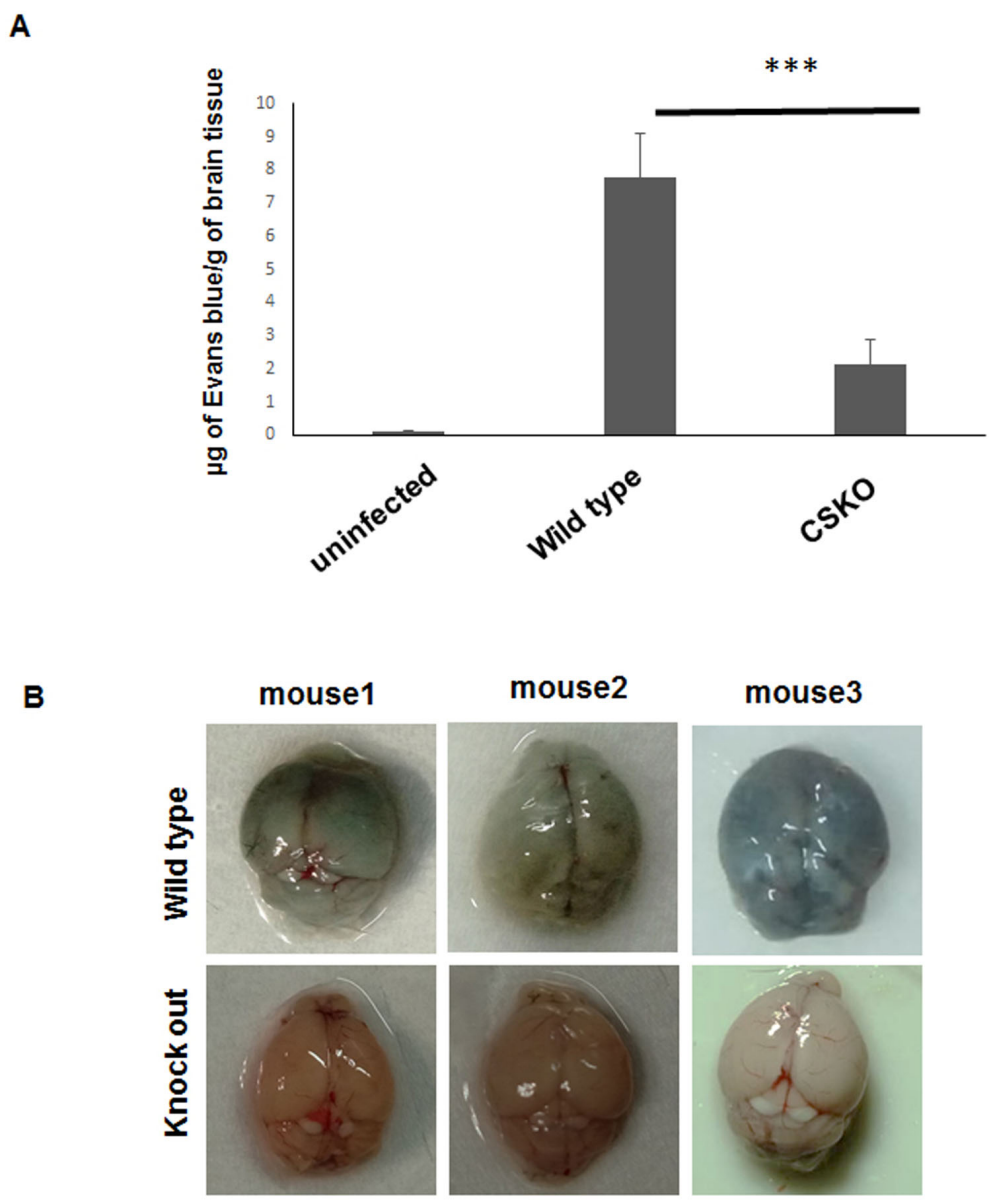
**Comparison of blood–brain barrier damage in wild-type and CSKO parasite-infected mice.** (A) Graph showing amount of Evans Blue leakage due to damage in blood brain barrier of mice infected with wild-type and CS knockout *P. berghei* (data are represented as mean±s.d. from two independent experiments). (B) Images of brains isolated from mice injected with Evans Blue showing entry of dye in wild-type parasite-infected mouse brain.

The level of blood–brain barrier damage was monitored through Evans Blue dye leakage assay. A higher level of blood–brain barrier damage was observed for mice infected with wild-type parasites with respect to uninfected control (Fig. 5A). The amount of leakage was approximately 8 µg dye per gram of brain tissue, whereas for the mice infected with the knockout parasite, significant reduction in dye leakage (***P<0.001) was observed, which was approximately 2 µg dye per gram of brain tissue, indicating less damage in the blood–brain barrier due to knockout parasite infection (Fig. 5A,B).

### SA-mediated induced expression of autophagy marker Atg8 in the *P. falciparum* 3D7 strain

**Fig. 6.**
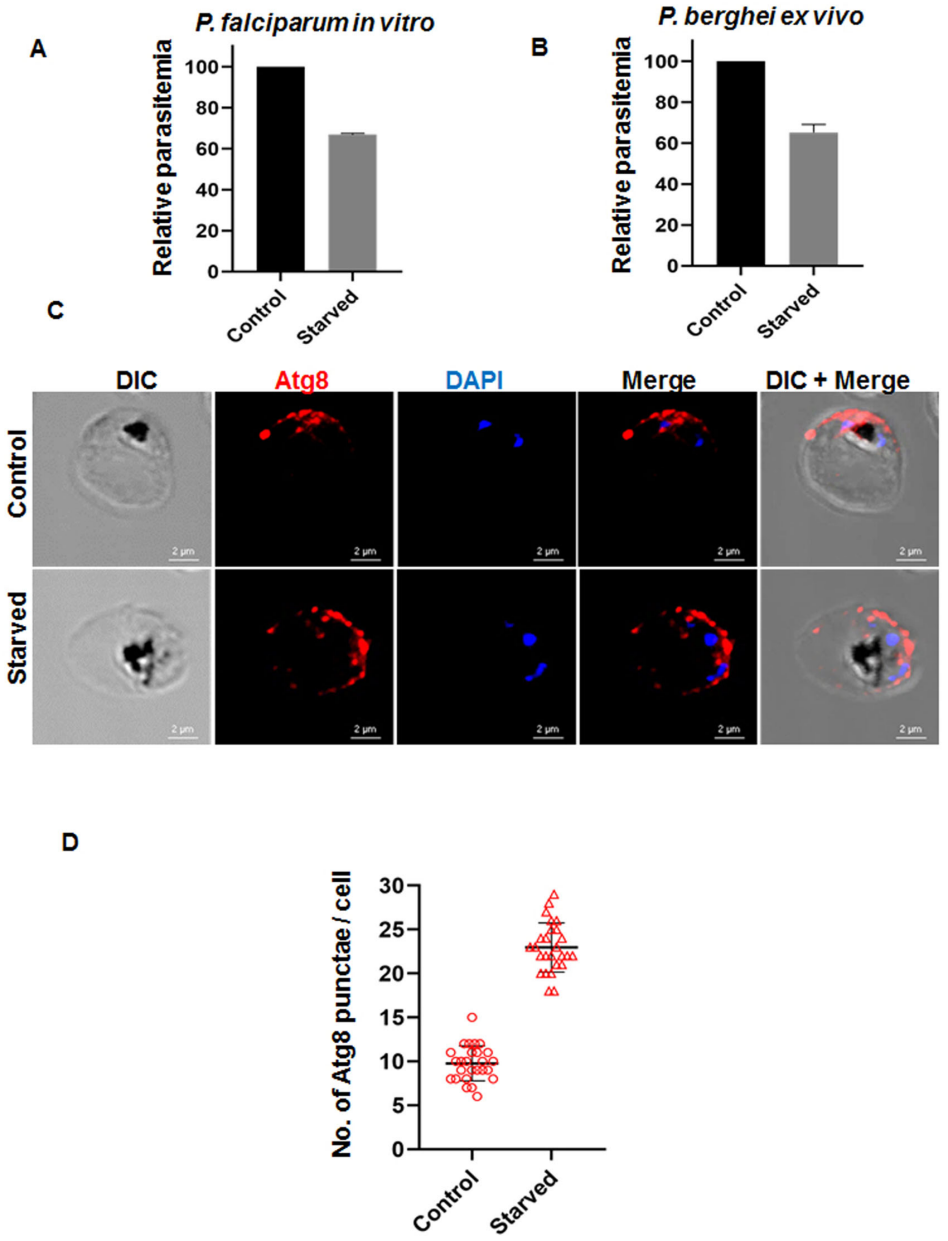
**SA mediated induced expression of autophagy marker Atg8 in *P. falciparum* 3D7 strain.** (A) *P. falciparum in vitro* starvation assay for 4 h. (B) *P. berghei ex vivo* starvation assay for 4 h. (C) IFA corroborating induction of autophagy in *P. falciparum* during 4 h starvation. (D) Representation of increase in the number of autophagic bodies (punctae) during starvation induced autophagy.

The ability of SA and its derivatives to induce autophagy is well explored in several other organisms (Pietrocola et al., 2018; Shamalnasab et al., 2018). However, the role of SA in Plasmodium remains elusive. Since the production of endogenous phytohormone SA by the malaria parasite has already been demonstrated (Matsubara et al., 2015), we aimed to study the mechanistic aspect of SA, whether SA encompasses the ability to induce autophagy within Plasmodium as well. To initially address whether parasites display starvation-induced autophagy we first analyzed the increase in the PfAtg8 expression and corresponding reduced invasion during starvation in our *in vitro* and *ex vivo* experimental conditions. Fig. 6A and B depict a reduction in the parasite invasion when incubated in nutrient depleted conditions under *in vitro* (*P. falciparum*) as well as *ex vivo* (*P. berghei*) systems. Further, the expression level of autophagosome marker PfAtg8 was altered under both the conditions within the parasite cytosol. Immunofluorescence assay revealed reduced Atg8 expression for control parasites indicating basal autophagy, while starved parasites showed induced autophagy with numerous small vesicles via PfAtg8 labeling resembling autophagosomes (Fig. 6C,D).

**Fig. 7.**
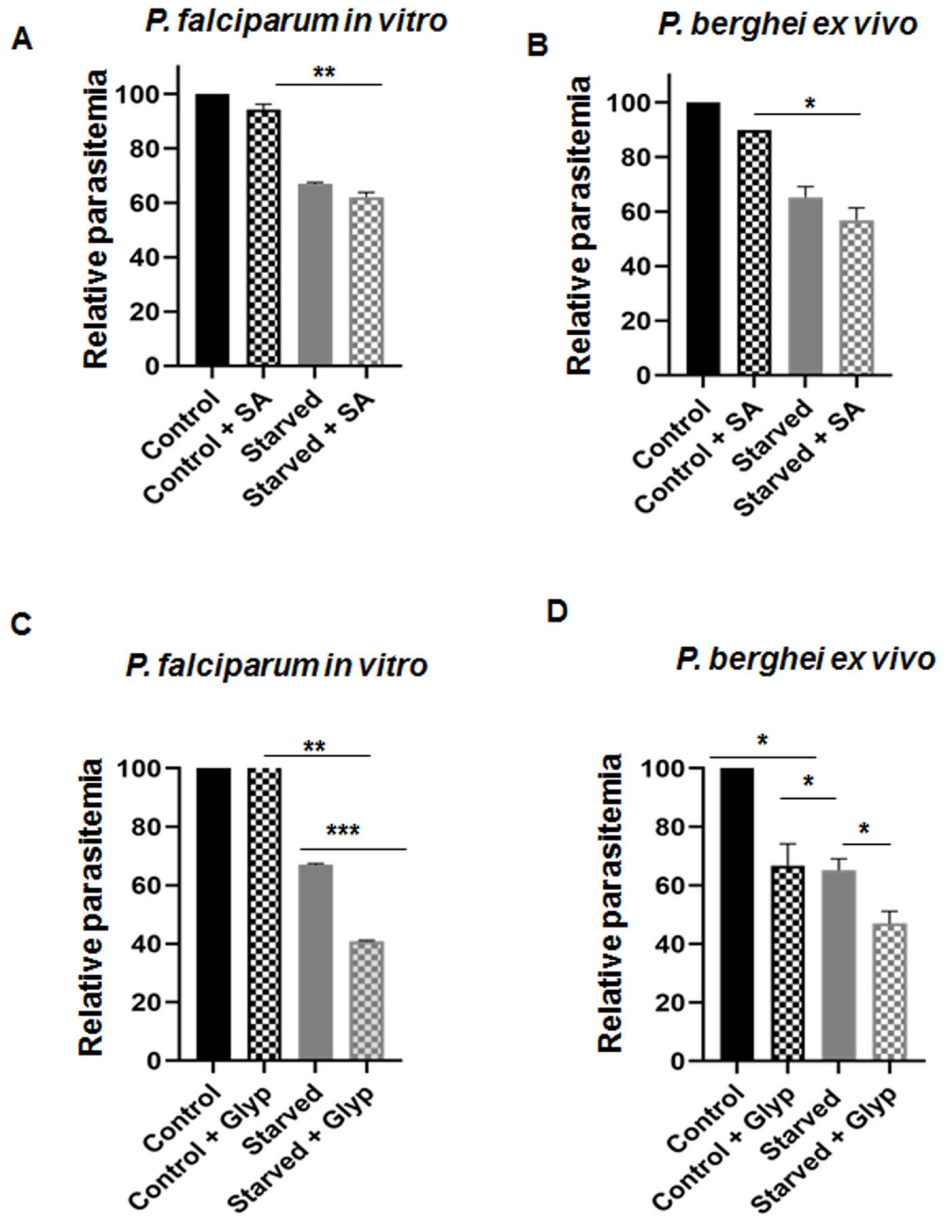
***Ex vivo* and *in vitro* starvation assay in *P. berghei* and *P. falciparum* showing effect of SA in parasite survival and importance of chorismate synthase.** (A) *P. falciparum in vitro* starvation assay in the presence and absence of SA during 4 h treatment. (B) *P. berghei ex vivo* starvation assay the presence and absence of SA during 4 h treatment. (C) *P. falciparum in vitro* starvation assay in the presence and absence of glyphosate (Glyp) during 4 h treatment. (D) *P. berghei ex vivo* starvation assay in the presence and absence of glyphosate during 4 h treatment. Data for the graphs of *in vitro* experiments (means±s.d.) were calculated from three independent biological experiments while for *ex vivo* experiments from two independent biological experiments.

Additionally, early/mid trophozoites when incubated with SA (1 mM) in a starved medium for 4 h led to a further reduction in parasitemia (Fig. 7A,B). However, a significant reduction in parasite invasion was not observed. Interestingly, the variation in the level of autophagy was evident by monitoring the autophagy induction via PfAtg8 expression by immunoblotting. PfAtg8 expression significantly increased upon starvation, while the expression of autophagosome marker was further augmented upon SA administration (Fig. S1A,B). These results demonstrate that although starvation invariably induces autophagy, the presence of SA may act as a probable trigger for inducing autophagy irrespective of the nutrient condition.

### Chorismate synthase: a key enzyme determining the parasite pathogenicity

Based on our *in vivo* studies, where mice infected with CSKO parasites yielded lesser pathogenesis, we postulated repression of SA synthesis resulted in reduced pathogenesis as compared to wild-type parasite-infected mice. Since we did not have an *in vitro* CSKO *P. falciparum* line, we utilized the herbicide Glyphosate as an inhibitor for subjugating SA synthesis. Glyphosate inhibits the enzyme EPSP synthase, which is upstream of chorismate synthase in the shikimate pathway. To validate the effect of SA inhibition *in vitro*, we treated the parasites under both nutrients replete and starved conditions with 5 mM glyphosate for 4 h at 37°C. Parasites when starved *in vitro* for 4 h showed a **∼**35% reduction in parasitemia, whereas treatment with glyphosate under starvation yielded ∼60% reduced parasitemia (Fig. 7C). As expected, *ex vivo* data also corroborated with a massive reduction in parasitemia in starved parasites under glyphosate treatment (Fig. 7D). Altogether, substantiating with our *in vivo*, *in vitro*, and *ex vivo* results, it is evident that inhibition of endogenous SA synthesis may relate to reduced pathogenesis due to reduced invasion. From these results, we may also speculate that inhibition of SA synthesis within the parasites may perturb the SA-dependent autophagic flux resulting in altered cellular homeostasis, hence, reduced pathogenesis.

## DISCUSSION

Cerebral malaria is a critical form of malaria affecting a large proportion of children residing in sub-Saharan African countries. Application of Aspirin or other SA derivatives in developing countries to reduce the febrile and inflammatory symptoms of cerebral malaria causes salicylate toxicity and prolonged inflammation followed by higher cerebral outcomes (also Reye's syndrome in some cases) (Aronoff, 2002). The mechanism behind this phenomenon is through inhibition of cyclooxygenase enzymes, especially Cox2, leading to the generation of PGE2, which is involved in reduction in inflammation by inducing anti-inflammatory cytokines at a later stage of inflammation. Lack of PGE2 induces prolonged activity of pro-inflammatory cytokines causing activation of immune cells and damage to host brain tissue (Ball et al., 2004). Also, SA increases the duration of activity of iNOS resulting into increased production of nitric oxide free radicles causing more damage (Clark et al., 2001). Furthermore, the parasite itself produces SA as a metabolite affecting host immunopathology in a prostaglandin mediated mechanism (Matsubara et al., 2015). These facts led us to establish a link between the SA-producing pathway of parasites and host pathobiology in relation with cerebral outcome.

The source of parasite SA, the shikimate pathway, is an interesting metabolic pathway for drug target identification as it is absent in mammals (Keeling et al., 1999). Earlier studies also show that the inhibition of a shikimate pathway enzyme EPSP synthase by the herbicide glyphosate caused growth inhibition of *P. falciparum* in *in vitro* culture (McConkey, 1999; McRobert et al., 2005). The terminal enzyme of this pathway, chorismate synthase is a possible target at it generates chorismate, the precursor of folate. SA is also generated from chorismate (Chen et al., 2009). Modulating the levels of this enzyme might alter the level of SA production in parasites thereby generating a new phenotype and its associated effect on the host. However, a recent study on *P. berghei* ANKA chorismate synthase knockout showed normal progression in mosquito and murine host (Choudhary et al., 2018) but the immunological, biochemical and pathological effects of the host upon infection of the knockout parasite are yet to be explored. In this work, we compared the immunological effects of wild-type and knockout parasites in murine host (Fig. 1A) using different methods. Application of SA in wild-type parasite-infected mice served as a positive control for increased cerebral outcome. Decreased cerebral outcome along with increased survival suggests that the absence of SA in parasites might influence the level of virulence of the parasite.

The possible mechanism of chorismate synthase knockout-mediated protection of the host might be facilitated through the downregulation of SA-dependent parasite autophagy. Interestingly, studies have already proven SA and its derivative salicylate to be a promising activator of AMP-activated protein kinase (AMPK). Salicylate activated AMPK further upregulates autophagic proteins and catabolic processes, thereby stimulating autophagy. Although the mechanism of autophagy and its integrated regulation is well illustrated in mammals, their presence in Plasmodium warrants further investigation. Recently, studies (Joy et al., 2018; Tomlins et al., 2013; Walker et al., 2013; Walker, 2013) have identified a majority of the autophagic proteins encoded within the genome, ensuring the presence of autophagy in the malaria parasite. Of note, Mancio-Silva (Mancio-Silva et al., 2017) demonstrated Plasmodium utilizes a putative serine/threonine kinase (KIN – with homology similar to mammalian AMPK) as a nutrient sensor, and may probably stimulate autophagy to modulate its pathogenesis. However, we suggest the signaling molecule triggering activation of KIN may plausibly be SA along with AMP/ADP. Analogous to mammalian AMPK activation cascade, in Plasmodium, the intrinsic SA synthesized by the parasite may act as a trigger or activator that binds and activates KIN, and its downstream targets favoring autophagy. Furthermore, KIN activation may also activate the sirtuins by increasing the cellular NAD+ level, initiating the interdependent positive feedback loop that regulates autophagy. However, the hypothesis of SA-dependent KIN-mediated autophagy is a conceivable mechanism and it needs to be explored further. The current study envisages the probable involvement of SA in the pathogenesis of malaria parasite via inducing the autophagic flux. With the parasite experiencing a nutrient-deprived and hypoxic niche upon sequestration by the endothelial cells during cerebral malaria, the probability of stimulation of autophagy is high. Based on our studies, the augmented Atg8 expression during starvation in the presence of SA strongly points towards SA as an inducer of autophagy, aggravating the pathogenesis. Moreover, the application of glyphosate (shikimate pathway blocker) resulted in reduced invasion of the parasites in the starved condition. Therefore, blockage of the parasite shikimate pathway may result in reduced synthesis of SA, thereby downregulating the SA-mediated autophagy and its associated pathogenesis in the form of severe cerebral outcomes. Additionally, the study also potentiates chorismate synthase as a new therapeutic target for combatting parasite virulence.

**Fig. 8.**
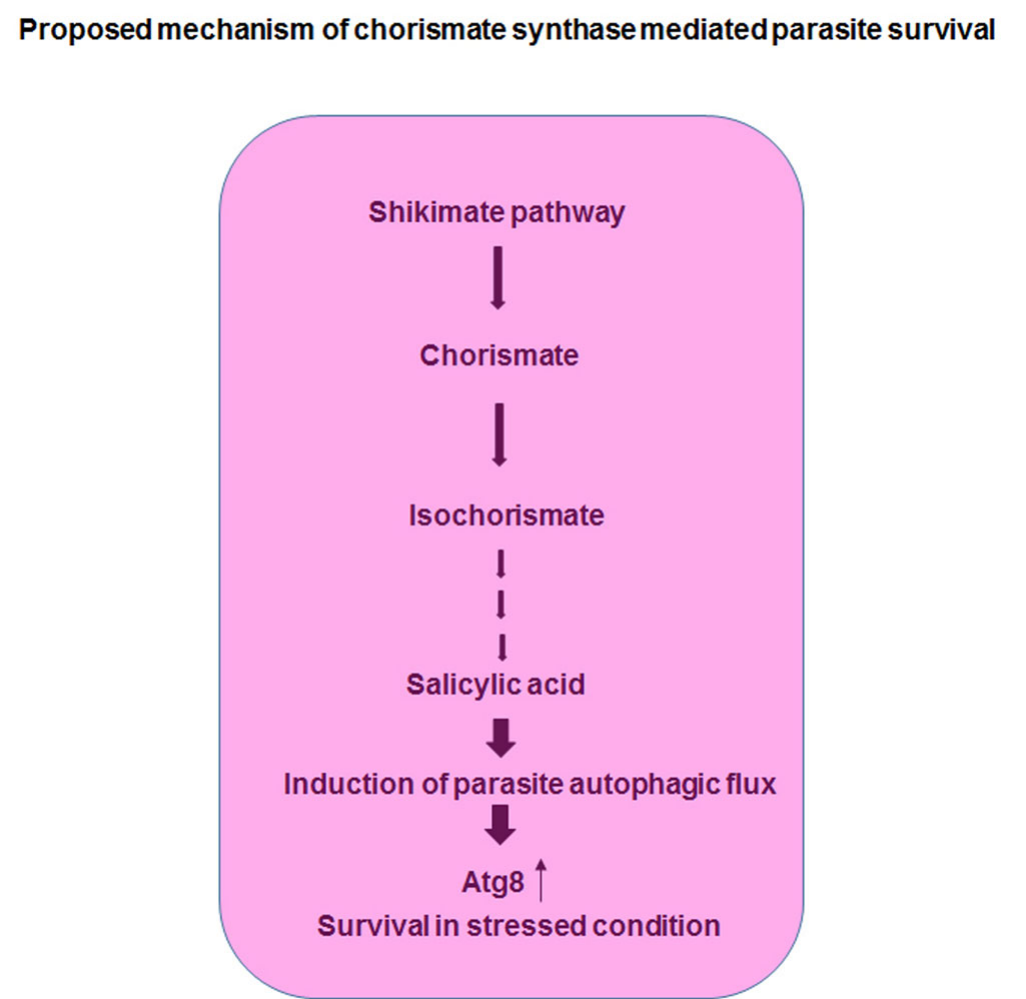
**Schematic showing the proposed mechanism of chorismate synthase and SA-mediated parasite survival.**

Overall, this study establishes the role of parasite chorismate synthase and SA on host immunopathology and disease manifestation. Moreover, a link has been determined between the immunomodulatory role of SA and parasite autophagy (Fig. 8). Further exploration in this area would facilitate in drug discovery against the disease.

## MATERIALS AND METHODS

### Infection of mouse with *P. berghei* ANKA wild type and Chorismate synthase mutant

C57BL/6 female mice of 6 weeks age were divided into four groups and each group consisted of eight animals. Malaria parasite *P. berghei* ANKA (obtained from BEI resources MRA-871) was passaged in two mice (both wild type and chorismate synthase mutant). Wild-type parasites were injected in the of two mice groups whereas *P. berghei* ANKA CSKO were injected in one group. Parasites (1×10^6^) were injected into each mouse intraperitoneally after diluting the infected mouse erythrocytes in 1× phosphate buffered saline (PBS) in a total volume of 200 µl. One group of mice was left as uninfected control. SA (Sigma-Aldrich, USA) was dissolved in a solvent mixture of DMSO, ethanol (Sigma-Aldrich) and PBS and injected in one mice group, infected with wild-type parasite at a dose of 100 mg/kg of mouse once a day until the tenth day after infection. The vehicle solvent mixture was injected to other groups of mice.

### Monitoring mouse parasitemia

Blood smears of the infected mice were prepared after collecting blood from the tail end of the mice. Smears were dried, fixed with methanol (Sigma-Aldrich), dried again and stained with Giemsa (Himedia, India). Parasitemia was determined by observing the stained smears under microscope at 100× magnification (Olympus Corporation, Tokyo, Japan).

### H&E staining of mouse brain tissue

Upon reaching a parasitemia of 20%, mice were anaesthetized by injecting a ketamine-xylazine cocktail (Sigma-Aldrich) intraperitoneally. Brains were dissected out and dipped in 4% paraformaldehyde (SRL chemicals, India). Sections of the brain tissue with 5 µm thickness were prepared through microtome after embedding of the tissue in paraffin. For staining, sections were deparaffinized by heating at 60°C for 15 min in order to melt the wax followed by washing with xylene (5 min), 100% ethanol (5 min) and 70% ethanol (5 min), respectively, and finally with water. Slides containing tissue sections were immersed in Mayer's Hematoxylin (Himedia, India) for 30 s followed by rinsing with water. Slides were again dipped in 1% Eosin Y for 30 s with agitation followed by washing with water. Tissue sections were dehydrated by washing with 95% ethanol, 100% ethanol and xylene, respectively, dried and mounted with mounting medium followed by covering the sections with cover slip.

### Evans Blue dye leakage assay

Evans Blue solution (2%) prepared in saline was injected intraperitoneally (4 ml/kg of mice) in mice of uninfected and infected groups. After 24 h of stain circulation, the mice were euthanized and the whole brains were removed. Mice brains were dipped in dimethyl formamide (Sigma-Aldrich) and incubated at 56°C overnight. Absorbance of the supernatant solvent was measured at 610 nm in a microplate reader (Varioskan Flash, Thermo Fisher Scientific, USA) and dye leakage was calculated through extrapolation from the standard curve prepared for varying concentrations of Evans Blue.

### RNA isolation from mouse brain tissue

Mice were euthanized after reaching 20% parasitemia and the whole brain was dissected out. Half of the brain tissue was dipped in Tri Reagent (Thermo Fisher Scientific) and stored at −80°C. Next day, the brains were taken out and homogenized uniformly into suspension using homogenizer (Thermo Fisher Scientific). The suspension was centrifuged at 13,000 RPM, 4°C for 15 min. Mouse brain RNA was isolated using the manufacturer's protocol from the supernatant obtained after centrifugation.

### cDNA synthesis from mouse brain RNA and qRT PCR of pro-inflammatory cytokines

Mouse brain RNA was quantified by NanoDrop (Thermo Fisher Scientific) and 2 µg of total RNA is converted to cDNA through reverse transcription using High Capacity cDNA Synthesis Kit (Applied Biosystems) according to the manufacturer's protocol. Expression patterns of Cox2, IL1β, TNFα and IFNγ were monitored through real-time PCR using Power Up SYBR green master mix (Applied Biosystems) and primers of the specified genes with mouse brain cDNA as template. Reactions were set up in triplicates using the following conditions: initial denaturation at 95°C for 5 min, annealing at 60°C for 30 s and extension at 72°C for 1 min with 40 cycles. The PCR was performed in Step-One Plus Real Time PCR instrument (Applied Biosystems) and fluorescence was measured after extension followed by melt curve analysis. Data were analyzed by Step-One software (Applied Biosystems).

### *P. falciparum* culturing and starvation

*P. falciparum* 3D7 line (derived from BEI resources MRA-102) was cultured in human O+ erythrocytes, at 37°C in complete RPMI 1640 medium with 0.5% Albumax and gassed with 5% CO_2_, 3% O_2_, and 92% N_2_. Parasite culture was synchronized at their ring stage via 5% sorbitol treatment. For further starvation based experiments, synchronized early trophozoite stage parasites (22–24 h.p.i) were cultured in complete medium or starved medium [Hank's Balanced Salt Solution (HBSS)] for 4 h duration at 37°C under the same gaseous condition.

### *P. falciparum in vitro* growth rate assay

To investigate the effect of starvation, trophozoite stage parasites were supplemented with complete (control) and starved medium for 4 h at 37°C. Whereas to determine the effect of SA, parasites were treated with 1 mM SA (Sigma-Aldrich) under similar complete and starved conditions for 4 h at 37°C. While for chorismate synthase inhibition *in vitro*, we mimicked the *in vivo* CSKO condition using EPSP synthase inhibitor glyphosate (Sigma-Aldrich). Parasites cultured in both complete as well as a starved medium were treated with 5 mM glyphosate for 4 h duration at 37°C. Post specific treatments, parasites were washed and cultured in complete medium for one cycle. Parasites were stained with EtBr solution and 100,000 cells were analyzed with FACS Fortessa (BD Biosciences, San Jose, CA, USA), using the software FlowJo. Forward light scatter (FSC)/side scatter (SSC) plot detected the samples simultaneously with EtBr fluorescence (FL1). Uninfected erythrocytes used for parasite culturing did not contain positive cells for the dye hence was used as negative control while untreated infected erythrocytes served as a positive control.

### *P. berghei ex vivo* starvation assay

Malaria parasite *P. berghei ANKA* (1×10^6^) was injected intraperitoneally in C57BL/6 mice and parasitemia was monitored by scoring the parasites in Giemsa stained smears regularly. When the parasitemia in blood reached 2%, blood was collected retro-orbitally in anti-coagulant containing tubes. Infected red blood cells were used for culturing after thorough washing and centrifugation at 500× ***g*** for 10 min. Un-infected red blood cells from healthy mice were used to maintain 1% hematocrit. Parasites were cultured in both complete (RPMI1640+20% FBS) as well as starved (HBSS) medium in the presence and absence of SA (1 mM), glyphosate (5 mM) for 4 h duration at 37°C under mixed gas conditions. Post specific treatments, parasites were washed and cultured in complete medium for 24 h. Following incubation, thin smears for each sample were methanol fixed and stained with Giemsa. Over 4000 red blood cells were scored for each slide to determine the parasitemia. Experiments were performed twice and relative parasitemia was determined based on the percent parasitemia calculated.

### Immunofluorescence Assay (IFA)

Synchronous *P. falciparum* parasites were cultured in complete and starved medium for 4 h at 37°C. Mid-trophozoites were fixed using fixative solution (4% paraformaldehyde and 0.0075% glutaraldehyde in PBS) for 30 min at room temperature. Parasites were washed twice with PBS and permeabilized using 0.1% Triton X-100 at room temperature for 3 min. Further parasites were washed and blocked with 3% bovine serum albumin (BSA) for 1 h at room temperature. The parasites were incubated with primary antibody in house rabbit raised anti-PfAtg8 (1:50) in 3% BSA for 1 h at room temperature followed by Alexa Fluor 594-conjugated goat anti-rabbit secondary antibody (Molecular Probes) at 1:300 dilution for 1 h at room temperature. Parasites were mounted over glass slide and mounted with ProLong Gold DAPI antifade (Molecular Probes). Confocal images were acquired using Nikon A1R MP+ multiphoton confocal microscope using a 100× oil immersion objective. Serial Z sections of each image were gathered and z-stack with best representations were illustrated in the figure. z-stack images were processed and deconvoluted for illustration via NIS elements software. Approximately 25 individual parasites were scored in control as well as starved conditions for determining the number of punctae per cell, respectively.

### Immunoblot analysis

For immunoblotting, pellets of synchronized trophozoite stage parasites grown in a complete and starved medium in the presence and absence of SA (1 mM) were collected. Parasites were separated from erythrocytes by saponin lysis (0.15% saponin in PBS) followed by washes with cold PBS. The parasites were suspended in SDS-PAGE sample buffer for 30 min at room temperature and sonicated with three pulses of 15 s each at 20% amplitude. Lysates were centrifuged at 16,000× ***g*** for 5 min and supernatant was collected. Protein samples were resolved on a 10% SDS-PAGE gel and transferred onto PVDF membrane (Millipore). The membranes were blocked with blocking buffer (5% skimmed milk in PBS-tween20) for 2 h at room temperature followed by overnight incubation at 4°C with primary antibody rabbit anti-PfAtg8 (1:100) in blocking buffer. The blots were washed and probed with HRP-conjugated anti-rabbit secondary antibody at dilution (1:2000) for 1 h at room temperature. The signals were developed with a western ECL kit (Bio-Rad) using Chemidoc (Bio-Rad). Images were processed for brightness and contrast by Photoshop (Adobe Systems Inc.).

### Data analysis

Parasitemia was assessed by flow cytometry or by microscopy using Giemsa-stained slides. Graphs were generated using GraphPad Prism 8. Student’s *t*-test for unequal variances was used to evaluate *P*-values. Data are from two (*ex vivo*) or three (*in vitro*) independent experiments done in duplicate.

### Compliance with ethical standards

All the *in vivo* and *ex vivo* experiments were carried out in accordance with the guidelines and regulations of Jawaharlal Nehru University and approved by institutional IBSC committee. Animal handling, parasite infection in animals and isolation of brain tissue were performed as per CPCSEA guidelines and approved by the Institutional Animal Ethics Committee (IAEC), Jawaharlal Nehru University, New Delhi.

## Supplementary Material

Supplementary information
