# Correction: Chorismate synthase mediates cerebral malaria pathogenesis by eliciting salicylic acid-dependent autophagy response in parasite

**DOI:** 10.1242/bio.059928

**Published:** 2024-05-28

**Authors:** Malabika Chakrabarti, Deepika Kannan, Akshay Munjal, Hadi Hasan Choudhary, Satish Mishra, Subhash Singh, Shailja Singh

**Affiliations:** ^1^ Host-Parasite Interaction & Disease Modelling Laboratory, Special Center for Molecular Medicine, Jawaharlal Nehru University, New Delhi 110067, India; ^2^ Department of Life Science, Shiv Nadar University, Noida, UP 201314, India; ^3^ Division of Parasitology, CSIR-Central Drug Research Institute, Lucknow 226031, India; ^4^ CSIR-Indian Institute of Integrative Medicine, Jammu, Jammu & Kashmir, India; ^5^ Current address: ICMR-Regional Medical Research Centre, Bhubaneswar, Odisha, India.

There was an error in *Biology Open* (2020) **10**, bio054544 (doi:10.1242/bio.054544).

Subhash Singh was added to the author list. Hadi Hasan Choudhary, Satish Mishra, Subhash Singh and Shailja Singh have reviewed and approved this correction; we were not able to contact Malabika Chakrabarti, Deepika Kannan, or Akshay Munjal. The corrected author affiliations and contributions are shown below; both the online full text and PDF versions of the paper have been corrected.

