# Supplementary material for: Chorismate synthase mediates cerebral malaria pathogenesis by eliciting salicylic acid-dependent autophagy response in parasite
Source: Biol Open. 2020 Dec 21;9(12):bio054544. doi: 10.1242/bio.054544 (PMC7774894; doi:10.1242/bio.054544)
Supplement: Supplementary information [file biolopen-9-054544-s1.pdf]

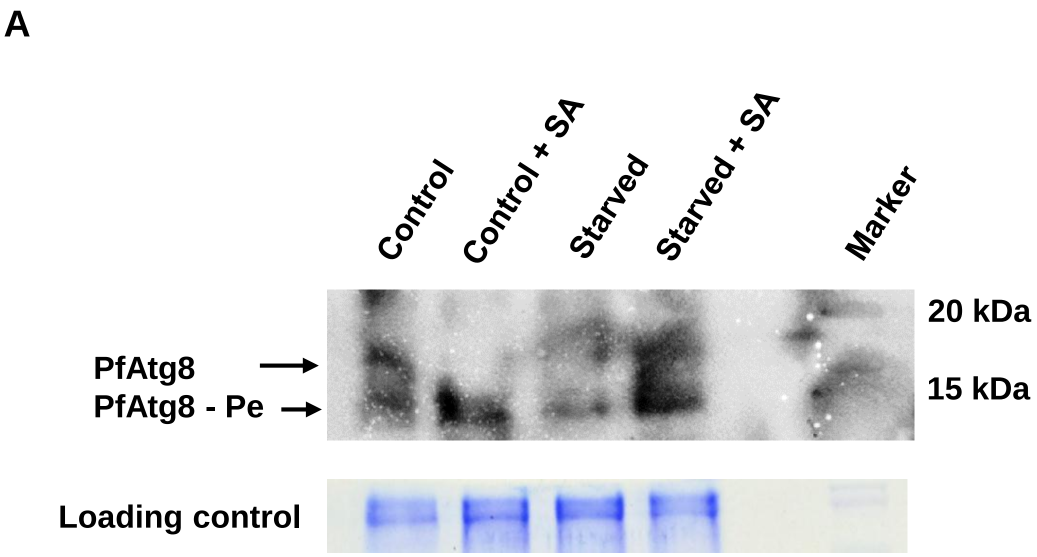

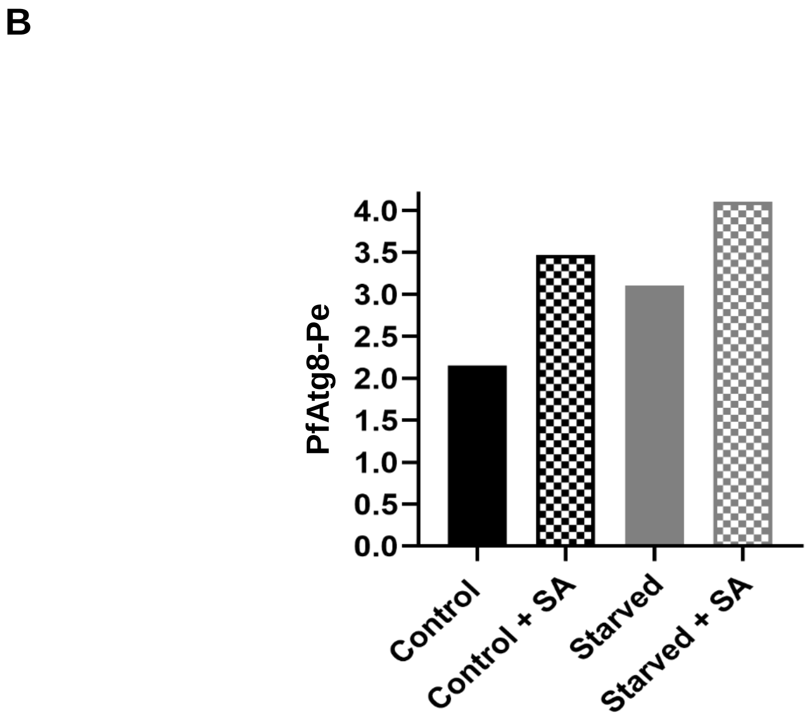

Fig S1. (A) Immunoblot-based quantification of lipidated autophagy marker PfAtg8-Pe levels in *P. falciparum* expressing the level of autophagy induction, **(B)** Comparison of the SA dependent induction of autophagy in complete (control) and starved condition.
